# Aerosol assisted synthesis of a pH responsive curcumin anticancer drug nanocarrier using chitosan and alginate natural polymers

**DOI:** 10.1038/s41598-023-46904-4

**Published:** 2023-11-08

**Authors:** Sepideh Asadi, Tayyebeh Madrakian, Mazaher Ahmadi, Miguel Ángel Aguirre, Abbas Afkhami, Seyed Sepehr Uroomiye, Fatemeh Ghaffari, Akram Ranjbar

**Affiliations:** 1 Faculty of Chemistry and Petroleum Sciences, Bu-Ali Sina University, Hamedan, 6517838695 Iran; 2 Department of Analytical Chemistry and Food Science and University Institute of Materials, Faculty of Science, University of Alicante, Alicante, Spain; 3 Department of Pharmacology and Toxicology, Faculty of Pharmacy, Hamadan University of Medical Sciences, Hamedan, Iran

**Keywords:** Drug delivery, Pharmaceutics

## Abstract

In recent years, several nanocarrier synthesis methods have been developed. In cancer therapy, the use of smart nanocarriers is of interest. Smart nanocarriers respond to their environment and can release their cargo in a controlled manner under the action of internal or external stimuli. In this work, we report on the development of an aerosol-assisted method for the synthesis of curcumin-loaded chitosan/alginate-based polymeric nanocarrier (CurNCs). A custom-fabricated multi-nebulizer system was utilized for the synthesis of CurNCs. The developed system comprises three main parts a sprayer, an electric heater tunnel, and a collector. Curcumin and chitosan solutions were sprayed using a pneumatic multinebulizer into the electric heater tunnel to form chitosan-curcumin assemblies. Then, the aerosol was guided into the collector solution containing sodium alginate and tri-poly phosphate aqueous solution for further cross-linkage. The synthesized CurNCs were characterized using TEM, DLS, and FTIR techniques. The TEM size of the nanoparticles was 8.62 ± 2.25 nm. The release experiments revealed that the nanocarrier is sensitive to the environment pH as more curcumin is released at acidic pH values (as is the case for cancerous tissues) compared to physiological pH. The curcumin content of the nanocarrier was 77.27 mg g^−1^ with a drug loading efficiency of 62%. The in-vitro cytotoxicity of the synthesized nanocarrier was evaluated against the MCF7 breast cancer cell line. The IC_50_ concentrations for CurNCs and curcumin were obtained as 14.86 and 16.45 mg mL^−1^, respectively. The results showed that while the empty nanocarrier shows non-significant cytotoxicity, the CurNCs impact the cell culture and cause prolonged cell deaths. Overall, pH-responsive curcumin polymeric nanocarrier was synthesized using a custom fabricated aerosol-based method. The method enabled fast and feasible synthesis of the nanocarrier with high efficiency.

## Introduction

Nowadays, targeted drug delivery has received much attention due to its many advantages. Most of the drugs used are composed of water-insoluble molecules^1^. Polymer drug delivery systems are used as useful tools for targeted drug delivery. These systems have been considered to deliver the drug to the desired location and prevent toxicity to other tissues, especially in the treatment of various types of cancer. Polymer systems are also widely used to reduce side effects and increase drug solubility. These systems can be designed in such a way that they are sensitive to an environmental or external stimulus and can release the drug at the desired location and cause targeted drug delivery^2^. Stimuli-responsive polymers or smart polymers are a group of polymers that can change their morphology and sometimes structure through physical or chemical changes and respond to external stimuli^3^. Various external factors and stimuli such as temperature, pH, CO_2_, light, electric and magnetic fields, and appropriate oxidizing or reducing agents are used^3–9^. One of the most used stimuli is pH. The extracellular pH in most cancer cells and tumors is acidic^10^.

pH-sensitive polymers act according to changes in the pH of their surroundings. For example, changes in the pH of the environment can lead to the release of the drug at the desired location. Most of these polymers have functional groups in their structure that can lose or gain protons in response to environmental pH. In addition, such systems increase the effectiveness and solubility of drugs in water and are more economical than the production of drugs with complex formulations. These systems also cause the controlled release of the drug. The use of these systems should be economical in addition to having reliable responses so that they can be chosen as a suitable alternative to the previous drug delivery systems^11^. Thus, the development of pH-sensitive polymer systems has received much attention^6,12–14^. These polymers can be of natural origin and can be synthesized.

Natural polymers have been widely used due to their abundance, biocompatibility, and biodegradability^15^. One of these widely used polymers is chitosan. Chitosan is a natural polysaccharide obtained from the skeleton of crustaceans, which is widely used in other fields in addition to medicine and drug delivery due to its unique properties^16^. Chitosan has many amine groups that are protonated and swollen at low pH, and the drug loaded in it can be released easily^17^. The sensitivity to pH has led to the widespread use of chitosan in drug delivery^18–21^.

Curcumin, which originates from the turmeric plant, has high therapeutic properties, but due to its low solubility in water alone, it cannot be used in drug delivery systems^14^. Curcumin has anti-inflammatory and antioxidant properties and is used in the treatment of various illnesses such as cancer, diabetes, and even Alzheimer's disease^22–24^. In 2022, Rajabzadeh-khosroshahi et al. synthesized a pH-sensitive nanocarrier based on a chitosan/agarose/graphitic carbon nitride composite. They used this hydrogel to release curcumin. Cytotoxicity of this hydrogel was investigated on a breast cancer cell line and this nanocomposite was used for the controlled release of curcumin with high performance and satisfaction^25^. In 2020, Gholamali et al. synthesized a carboxymethyl chitosan/polyvinyl alcohol-based hydrogel containing silver nanoparticles. This hydrogel is sensitive to pH and the swelling was investigated at pH 2.1 and 7.4 (environment similar to stomach and intestine) and antibacterial activity was proved^26^. In our recently published paper, we utilized chitosan for the synthesis of pH-sensitive curcumin-loaded chitosan nanocapsules^27^.

Generally, the use of synthesis or purification methods of nanomedicines and nanomaterials is associated with high consumption of resources, and the low yield of the product and the difficulty of controlling the size of the particles and the final composition of the product are among the challenges that prevent the production of these systems on a larger scale or for commercial use^28–34^. In general, size control is an important issue in drug delivery. The aerosol-based technique is one of the most powerful in nanomaterial synthesis. These techniques incorporate some physical processes and chemical reactions^35^. Aerosol particles can be created by nebulizing liquids with a specific chemical formula using a variety of techniques, including ultrasonic nebulization, spray pyrolysis, pneumatic nebulizers, and electro-synthesis nebulization^36,37^. The importance of nebulization in particle size determination cannot be overstated. During the nebulization process, the original precursor is fed through a specialized nebulizer to produce a huge number of nano/micro-sized droplets. Depending on the droplet size requirements, centrifugal, ultrasonic, electrostatic, and gravity forces can be used to improve the nebulization technique^38^. In this process, a liquid nebulization nozzle turns the liquid supply into small droplets using nebulizers such as rotary disk, pneumatic, ultrasonic nozzle, and hydraulic friction nebulizers. The nebulized droplets' solvent evaporates, causing the droplets to freeze and form nanomaterials^39,40^. Pneumatic nebulizers include pneumatic concentric nebulizers, pneumatic nonconcentric nebulizers, thermospray nebulizers, and pneumatic micronebulizers^41^. In pneumatic nebulizers, a high-velocity gas stream impinges on a relatively low-velocity liquid stream, and the kinetic energy of the pressurized gas is used to generate liquid surface (i.e. the aerosol). In pneumatic concentric nebulizers, the liquid capillary is concentric to the gas capillary. The liquid–gas interaction is produced when the high-velocity gas stream tangentially enters into contact with the liquid. The geometry of this interaction is annular. This is the most common nebulizer used in atomic spectrometry, as it is easy to handle, relatively low-cost, and has adequate robustness^42^. To simultaneously nebulize several different solutions, multinebulizer devices have been introduced. To this end, different approaches to achieving simultaneous nebulization have been developed such as using a modified nebulizer or using two of more nebulizer arrangements^41^. In our work, two independent pneumatic nebulizers were utilized for the simultaneous nebulization of precursor solutions.

As a result, the main aim of this research is to develop an aerosol-assisted strategy for the synthesis of chitosan-alginate nanocarriers for use in drug delivery of curcumin as a low water-soluble model drug. The setup used for the synthesis is custom-fabricated and can be easily fabricated and scaled up. The synthesis of drug-free nanocarriers as well as nanocarriers containing curcumin was carried out in a simple and one-step process.

## Experimental section

### Reagents and apparatus

All the chemicals used were of analytical reagent grade or the highest purity available and were purchased from Merck Company (Darmstadt, Germany). Aqueous solutions of chemicals were prepared with deionized water. Curcumin (CUR) and low molecular weight (molecular weight: 50,000–190,000 Da; viscosity: 20–300 cP, 1 wt. % in 1% acetic acid) chitosan (75–85% deacetylated) and sodium alginate powder were purchased from Sigma-Aldrich Company (St. Louis, MO, USA). All glassware was soaked in dilute nitric acid for 12 h and.

then thoroughly rinsed with deionized water. Sodium dihydrogen phosphate, sodium hydrogen phosphate, and sodium chloride salts were used to prepare phosphate saline buffer (0.1 mol L^−1^). In this work, Rorea, Korea 40 kHz ultrasonic bath and Hettich Instruments, LP, Germany MIKRO 220 microcentrifuge were used. A model IDEX/ISMATEC ISM 4208 peristaltic pump was used. The absorbance of the samples was measured with a single beam UV/Vis spectrophotometer WPA model Lightwave and a 1 cm quartz cell. GR-202 model scale with four decimal places accuracy was used to weigh the materials. To measure the pH of the solutions, a pH meter model 713 made by the Swiss Company Metrohm was used. Fourier-transform infrared (FT-IR) analysis was performed utilizing an FT-IR spectrometer (Model Spectrum GX, Perkin Elmer Company) in the 4000–400 cm^−1^ area using the KBr pellets technique to investigate the chemical structures of the synthesized nanomaterials. To visualize the structure and size of the synthesized nanocarrier, a transmission electron microscope (TEM) with a TEM Philips EM 208S model was used. The power nanoparticles were dispersed in methanol using a sonication water bath. Then one drop of the dispersion was cast on the TEM grid. The solvent was evaporated and the grid was analyzed using the TEM instrument. Dynamic light scattering (DLS) method using the Zetasizer Nano ZS Malvern instrument was used for the evaluation of the size distributions of synthesized nanoparticles. Phosphate buffer solution (PBS) was used to adjust the pH of the release mediums. The MCF7 breast adenocarcinoma cell line was obtained from the Institute Pasteur, Iran. Dulbecco's Modified Eagle Medium (DMEM) cell culture medium and a penicillin and streptomycin antibiotic mixture were obtained from Kiazist Company (Hamedan, Iran). Fetal bovine serum (FBS) was purchased from Gibco (Thermo Fisher Scientific). A 3-(4,5-dimethyl-2-thiazolyl)-2,5-diphenyl-2-H-tetrazolium bromide (MTT) assay to evaluate the cytotoxicity effect of the nanocarrier on MCF7 cells was performed in the School of Pharmacy at Hamadan University of Medical Sciences, Hamadan, Iran.

### Design of the developed multi-nebulizer-based synthesis system

**Figure 1:**
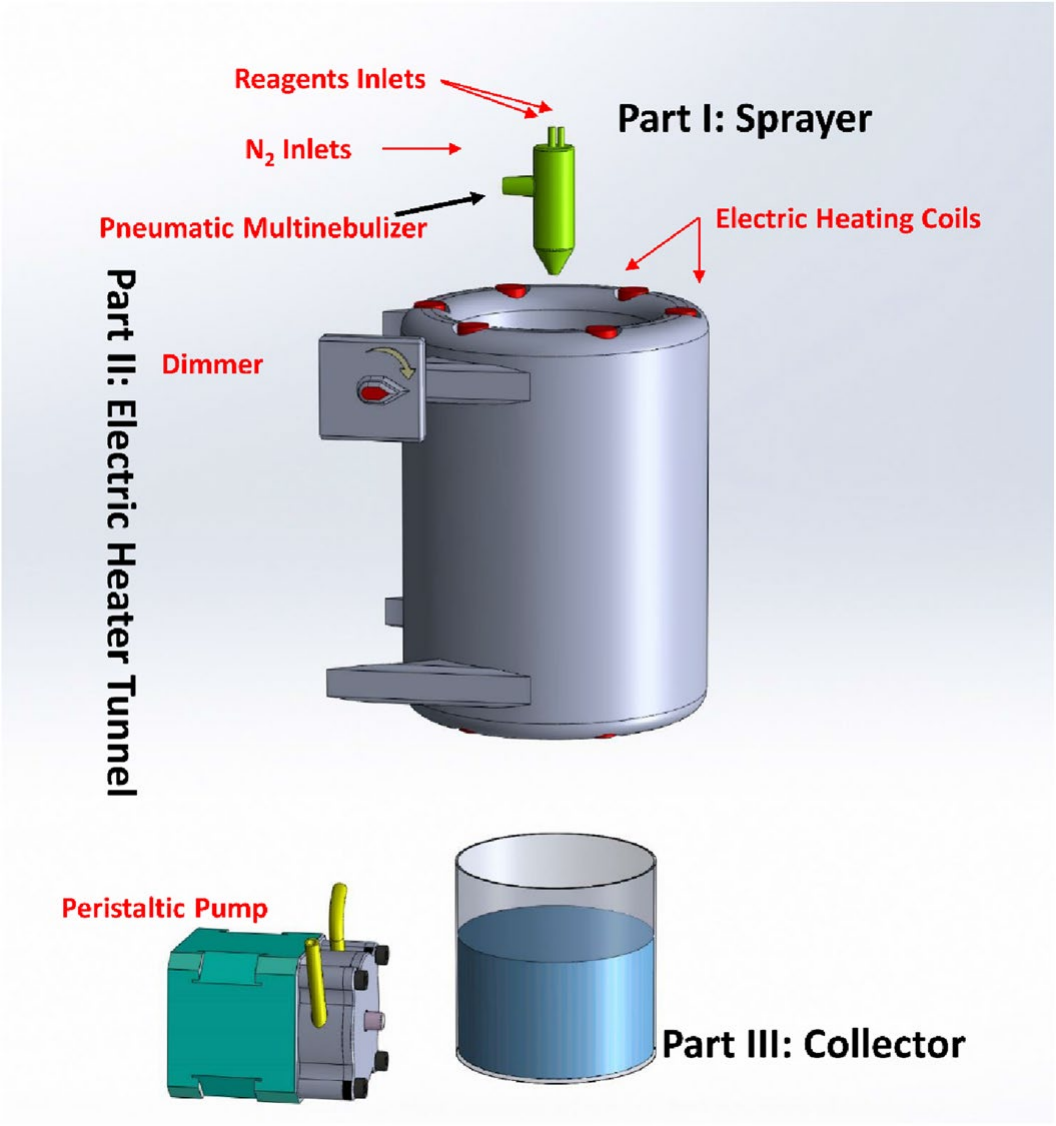
Schematic illustration of the developed multinebulizer-based aerosol-assisted synthesis system.

Figure 1 shows a schematic representation of the developed system. The developed system is made up of three major components:

- **Sprayer (Part I)** A peristaltic pump and a handmade glass concentrate pneumatic multinebulizer with two parallel untreated fused silica capillaries comprise this component^43^. The carrier gas for the creation of aerosols of the chemicals fed into the nebulizers was high-purity nitrogen gas.
- **Electric heater tunnel (Part II)** Tunnel with an electric heater. This component is made up of six tungsten filaments surrounded by cylindrical stainless-steel plates. The tunnel temperature was also set using a dimmer. An infrared thermometer was used to measure the tunnel temperature. The multinebulizer aerosol passes into the heated tunnel for the additional reaction of the precursor reagents of the produced nanoparticles.
- **Collector (Part III)** This part consists of a beaker containing a cross-linker agent. The cross-linker solution is stirred using a magnetic stir bar.

### Aerosol-assisted synthesis of curcumin-loaded chitosan/alginate nanocarrier (CurNCs)

Three solutions were prepared for the synthesis of CurNCs. The solution I contained 2 mg of curcumin powder dissolved in 10 ml of ethanol. Solution II contained a solution of chitosan (4 mL of 1% (w/v) chitosan solution in 4% (v/v) acetic acid + 6 ml of deionized water). Cross-linker solution III, which contains sodium alginate solution (200 mg of tri-poly phosphate (TPP) in 20 ml of deionized water + 4 ml of 0.5% (w/v) sodium alginate solution). Solution I and II were simultaneously nebulized by the pump (flow rate = 0.02 mL s^−1^). Two aerosol streams collide and interact at the intersection (tip of the nebulizer) and during passing through the electric heater tunnel (Temperature = 40 °C). Then, the mixed aerosols are guided to solution III and react further with magnetic stirring for 30 min. The resulting solution was centrifuged and then washed three times with deionized water to remove unreacted precursors and the sediment obtained was dried under vacuum and collected for the next steps. The synthesis of empty nanocarrier (ENCs) was performed using the same procedure but solution I here contains 10 ml of ethanol without curcumin.

### In vitro* release studies*

To investigate the performance of the synthesized nanocarrier and its application in targeted drug delivery, the drug release process from the synthesized nanocarrier was investigated at two pH levels (7.4 and 5). For this purpose, first, 11 mg of curcumin-loaded nanocarrier (77.27 mg_curcumin_/g_nanocarrier_) was weighed. Then, for each pH, a mixture containing 2 ml of ethanol and 3 ml of PBS was added. It is worth mentioning that a mixture of ethanol and PBS was utilized for the release experiment due to the low solubility of curcumin in water at neutral and acidic pH values. Then, to check the drug release process, the nanocarrier and the mixture of ethanol and buffer were placed in a shaker machine at a temperature of 37.4 °C and an rpm = 60. Then, with specific time intervals, the absorbance intensity of the solutions at the wavelength of 432 nm was recorded by a UV–Vis spectrophotometer (calibration equation: A = 31.516C (mg mL^−1^) + 0.0016, R^2^ = 0.999, dynamic linear range: 0.8–40 µg mL-1).

### In-vitro cytotoxicity assay

MCF7 breast cancer cells were used as a model to test the cytotoxicity of the synthesized CurNCs by MTT assay. The culture medium containing FBS was prepared by mixing DMEM culture medium with 10% FBS and 1% penicillin–streptomycin. First, the cells were grown in flasks with filter lids in a DMEM culture medium containing 10% FBS in an incubator at 37 °C and 5% CO_2_ until reaching 80% confluence. The cells were then passaged. After reaching a sufficient number, 10,000 cells per well were seeded in a 96-well plate. Thus, after 24 h, they were treated with different concentrations of curcumin and nanocarriers. It should be noted that the first row as the control row contained only the culture medium (control group). After certain incubation times, their survival rate was evaluated using the MTT cell viability assay^14^. To perform the MTT assay, 20 µl of the filtered solution were added to each well. Then, after 3 h, the wells were emptied and 100 µl of dimethylsulfoxide (DMSO) were added to each well to dissolve the formed formazan deposits. The absorbance was then read and recorded using an ELISA reader at 570 nm.

## Results and discussion

### Characterization of the nanocarrier

**Figure 2:**
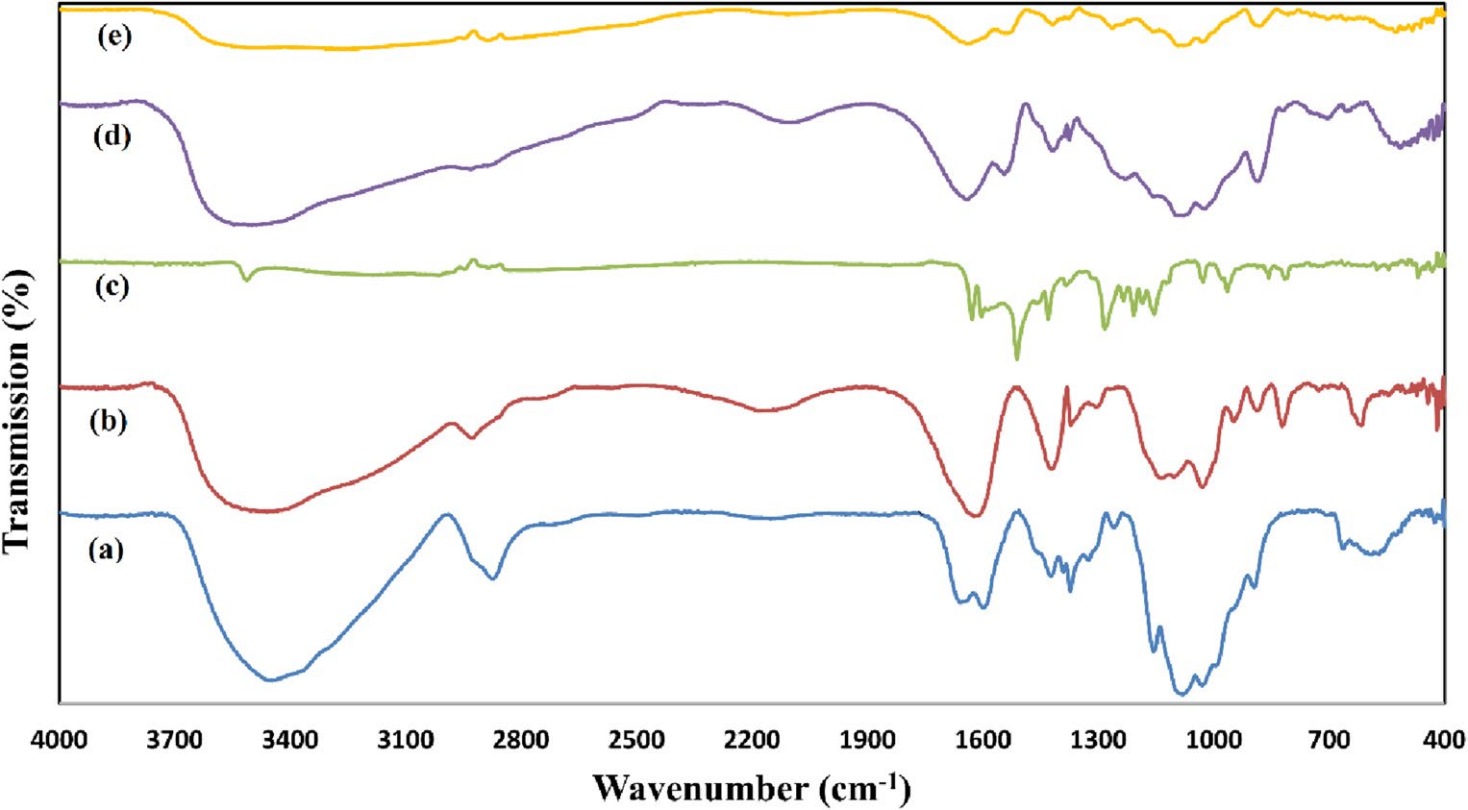
The FT-IR spectra of chitosan (**a**), sodium alginate (**b**), curcumin (**c**), synthesized chitosan/alginate nanocarrier without the drug (ENCs) (**d**), and synthesized curcumin loaded chitosan/alginate nanocarrier (CurNCs) (**e**).

Figure 2 shows the FT-IR spectra of the synthesized nanocarrier and precursors. To ensure the successful synthesis of the nanocarrier, the FT-IR spectra of the samples were obtained at different stages and compared with each other. Figure 2A is the FT-IR spectrum of pure chitosan powder. The peak at 3446 cm^−1^ is related to the amine and hydroxyl groups of chitosan. The peak at 2870 cm^−1^ is related to C–H stretching vibration, 1650 cm^−1^ is related to C = O, 1590 cm^−1^ is related to NH_2_ vibration, 1370 cm^−1^ is related to COOH vibrations, and 1079 cm^−1^ is related to C–O stretching mode in chitosan. Figure 2B is the FT-IR spectrum of pure sodium alginate powder. The peak at 3450 cm^−1^ is related to O–H stretching vibrations in alginate. C–H stretching vibrations were observed at 2930 cm^−1^. The peak at 1028 cm^−1^ is related to C–O–C stretching and 1622 and 1422 cm^−1^ are related to symmetric and asymmetric stretching vibrations in COO^−^. Figure 2C is the FT-IR spectrum of curcumin powder. The peak at 1627 cm^−1^ is related to C=C symmetric aromatic ring stretching, the peak at 1510 cm^−1^ is related to C=O of the benzene ring, 1429 cm^−1^ is related to C–H bending vibration, 1282 cm^−1^ is related to aromatic C–O stretching vibrations, and 964 cm^−1^ is related to C–O–C stretching vibrations in curcumin^44–47^.

Figure 2D shows the FT-IR spectrum of chitosan/alginate nanocarrier without drug. This spectrum shows a state of two peaks of pure chitosan and alginate. Due to the electrostatic interaction of the amine groups of chitosan with the carboxylate groups of alginate, The peak at 3446 cm^−1^ corresponding to the amine and hydroxyl groups of chitosan and the peaks at 1622 and 1422 cm^−1^ corresponding to the carboxyl group in alginate have shifted to 3528, 1640 and 1543 cm^−1^, respectively and their intensity has also decreased^48^. Figure 2E shows the FT-IR spectrum of the chitosan/alginate nanocarrier containing curcumin. This peak is very similar to the peak related to the nanocarrier containing chitosan/alginate, but due to the interaction of curcumin with the functional groups of the nanocarrier, shifts are observed. For example, the peak related to the benzene ring at 1510 cm^−1^ in curcumin powder has shifted to 1545 cm^−1^ in the nanocarrier containing curcumin. The peak related to stretching vibrations in benzene in curcumin powder shifted at 1627 cm^−1^ and reached 1637 cm^−1^ in the nanocarrier containing curcumin. Some peaks are also observed in the nanocarrier containing curcumin, whose intensity is reduced, which can be caused by the overlap of the peaks. For example, the intensity of the peaks at 1637 and 1545 cm^−1^ corresponding to the chitosan/alginate nanocarrier containing curcumin, respectively, is reduced compared to the chitosan/alginate nanocarrier and curcumin powder, which can be caused by the overlap of the peaks^49^. The shift of peaks and relative decrease in intensity might be due to the interaction of chitosan and alginate functional groups with curcumin suggesting the successful synthesis of nanocarrier containing curcumin. However, to confirm the interactions, further analysis like NMR should be performed.

**Figure 3:**
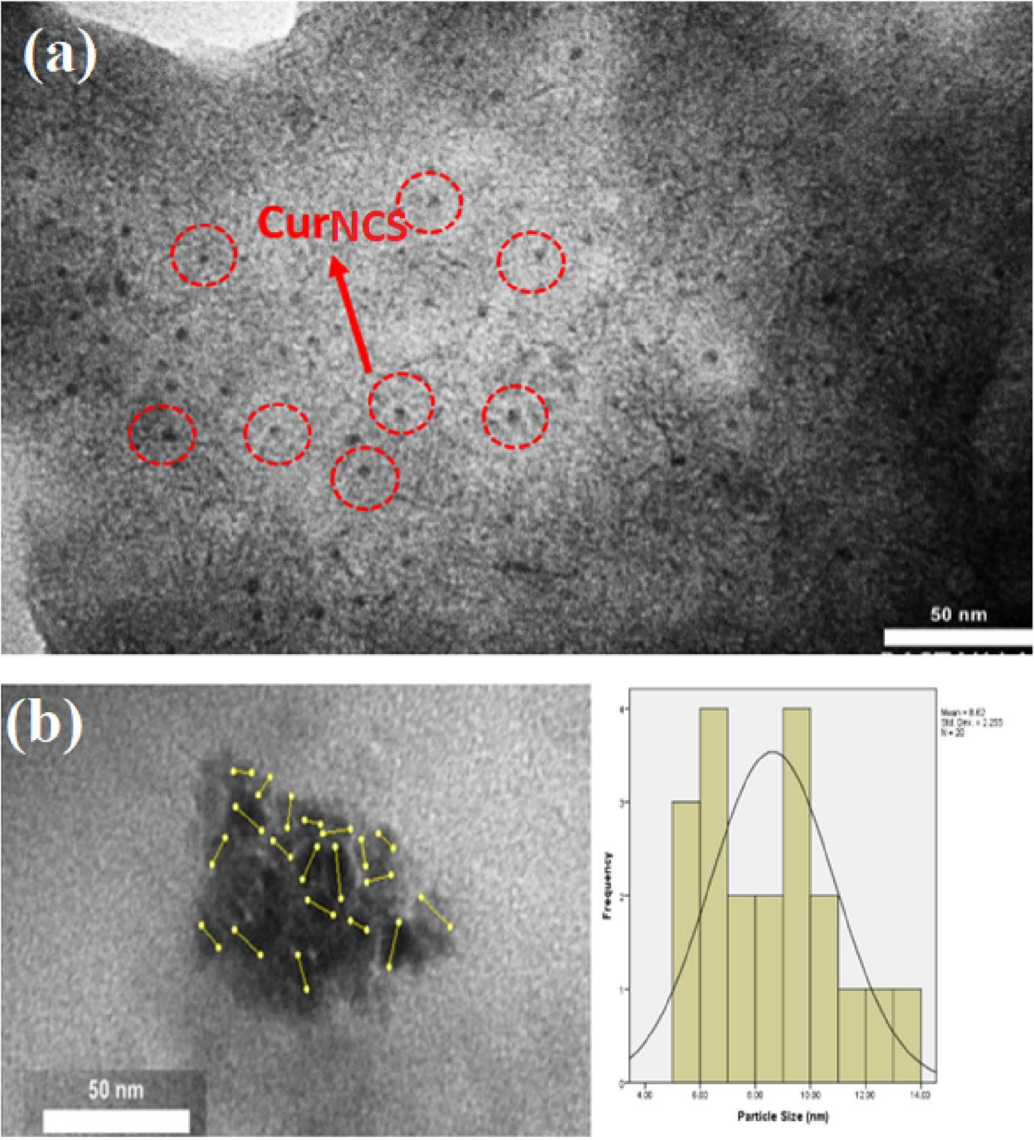
The TEM image (**a**) and particle size histogram (**b**) of the synthesized CurNCs.

A transmission electron microscope was used to determine the size of the CurNCs. Figure 3 shows the relevant TEM images and particle size histogram for the nanocarrier containing curcumin. Based on the obtained images and results, the average diameter of the synthesized nanocarrier is about 8.62 ± 2.25 nm, which indicates the small size of the synthesized nanocarrier, which can be well used in targeted drug delivery.

**Figure 4:**
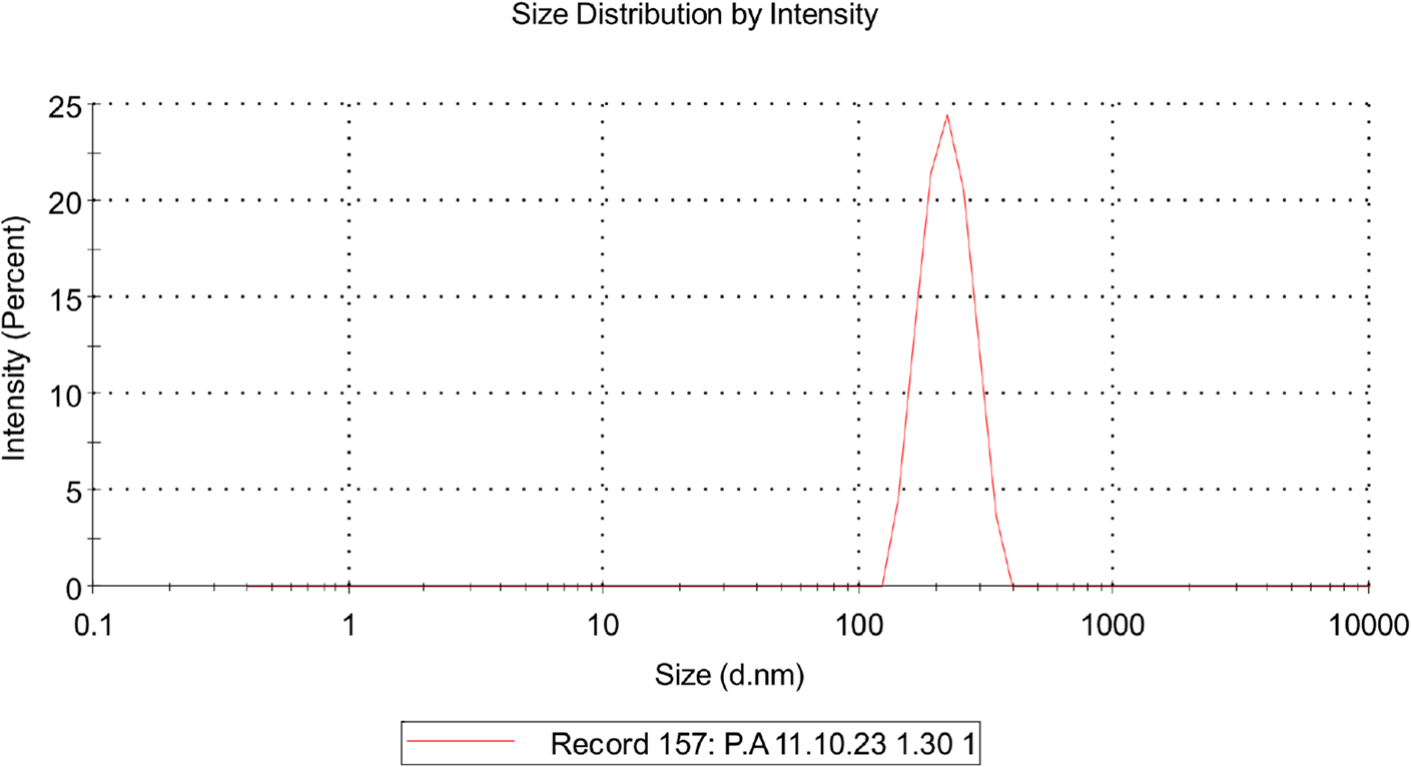
The DLS size distribution of CurNCs.

The average size of CurNCs using the DLS method was estimated to be 223 ± 48 nm (Fig. 4). The estimated size for CurNCs using the DLS method is larger compared to the TEM analysis due to the swelling and agglomeration and of the nanoparticles in the test solution.

### Investigation of loading capacity and loading efficiency of curcumin in chitosan/alginate nanocarrier

By measuring the absorbance of curcumin in the synthesized nanocarriers, it is possible to calculate the amount of drug loading capacity, mg of drug placed in the nanocarrier, and the percentage of loaded drug. First, the weight of the nanocarrier in the synthesis must be determined. For this purpose, after the synthesis, the weight of the nanocarrier was obtained with a balance. Then, one gram of the synthesized nanocarrier was weighed and made up to 2 ml using a solvent mixture of 1 mol L^−1^ hydrochloric acid and acetone with a ratio of 70 to 30^27^. To completely dissolve the nanocarrier in the solvent, the nanocarrier was placed in an ultrasonic for 15 min and then the suspended material was separated using a centrifuge for 5 min. In the end, the absorbance of the supernatant solution was measured using a UV–Vis spectrophotometer. The maximum absorbance of curcumin is at the wavelength of 432 nm. Then, by drawing the calibration curve^50–53^ for curcumin (calibration equation: A = 31.516C (mg mL^−1^) + 0.0016, R^2^ = 0.999, dynamic linear range: 0.8–40 µg mL^−1^) and using the absorbance intensity of the synthesized nanocarrier and according to the amount of drug dissolved in the first stage of synthesis, mg of drug placed in the nanocarrier, the amount of drug loading capacity, and the percentage of loaded drug using Eqs. 1 and 2 were calculated.

1 $$\mathrm{Drug\; loading\; capacity}(\frac{\mathrm{mg}}{\mathrm{g}})=\frac{\mathrm{The\; weight\; of\; the\; drug\; in\; the\; nanocarrier}(\mathrm{mg})}{\mathrm{The\; weight\; of\; the\; nanocarrier}(\mathrm{g})}$$

2 $$\mathrm{\%Drug\; loading\; efficency}=\frac{\mathrm{The\; weight\; of\; the\; drug\; curcumin\; in\; the\; synthesized\; nanocarrier}}{\mathrm{Initial\; Curcumin\; Drug\; Weight}}\times 100$$

As a result, using the calibration equation and Eqs. 6 and 7, the amount of curcumin drug in 1 mg of nanocarrier was obtained as 0.077 mg. According to Eq. 6, the loading capacity of the curcumin drug was found to be 77.27 mg per gram of nanocarrier. The drug loading efficiency of the curcumin drug in the synthesized nanocarrier was 62% according to Eq. 2.

### Investigating the effect of pH on the release process of curcumin drug

**Figure 5:**
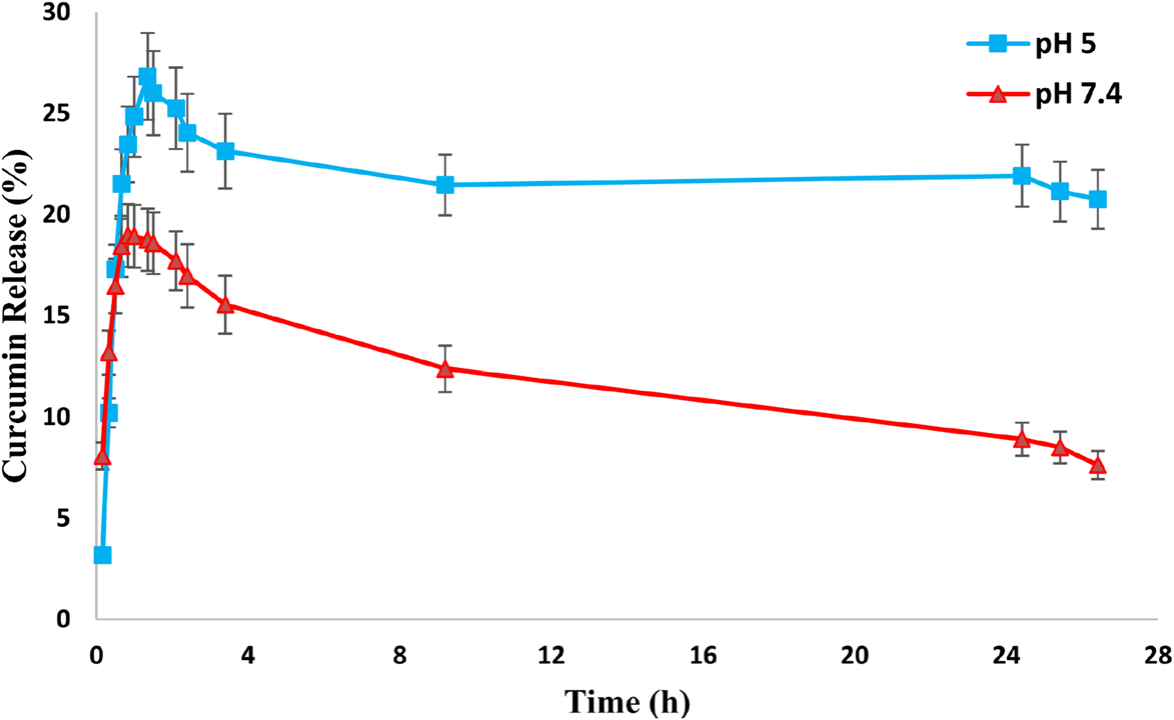
The release profile of curcumin drug from the CurNCs in 5 ml of PBS buffer solvent mixture and ethanol in a ratio of 60:40 at pH 7.4 and pH 5 at a temperature of 37.4 °C and rpm = 60.

The release studies results (Fig. 5) indicated that the release rate of the curcumin drug was higher in acidic conditions compared to normal physiological pH. After some time, in both graphs, the absorbance of the solutions is almost constant and decreases slightly, which might be due to the re-adsorption of the drug. As obtained from the results, the synthesized nanocarrier is sensitive to pH and the release rate of the model drug is different with time at pH 7.4 and 5. The release rate of curcumin drug at pH = 5 is higher than 7.4. This indicates the fact that the synthesized nanocarrier can be used in drug delivery to cancer tissues and cells considering that the normal physiological pH of the body is 7.4 and the pH of cancerous tissues is acidic^10^.

### Curcumin release profiles from CurNCs

To find out the possible mechanism of curcumin drug release from CurNCs, curcumin release data at pH 5 and 7.4 were obtained with different kinetic models by the Nonlinear Least Squares Regression (Curve Fitter) program, and the value of correlation coefficient (R^2^) and Root Mean Square (RMS) were obtained. The kinetic parameters of curcumin release related to different kinetic models are summarized in Table 1. According to the information obtained from Table 1, the values of R^2^, speed constant (k), and RMS have been compared for different models. The closer the R^2^ values are to 1, the better the fit of the experimental data of curcumin release with that kinetic model. When the R^2^ values are close to each other, comparing the RMS values can help to compare the data from different kinetic models. The lower the RMS value, the better. According to the obtained results; Due to the larger R^2^ values, curcumin drug release at both pH values followed the First order model. According to the first-order equation, the reaction speed is proportional to the drug concentration. According to Fick's law of diffusion, drug molecules move from a place with a higher concentration to a place with a lower concentration to reach an equilibrium state, and the rate of diffusion is proportional to the concentration gradient in the system^54^. The value of k indicates the release rate of curcumin. According to the obtained results, the value of k at pH = 5 is greater than 7.4; which indicates that the release is faster at pH = 5 than 7.4. As a result, considering the acidity of cancer cells, this nanocarrier can be used to deliver drugs to cancer cells. On the other hand, these results confirm the presence of amine groups in chitosan. The amine groups are protonated and swelled by exposure to acidic environments and release the encapsulated drug more and faster. According to the data obtained from fitting the release of curcumin according to the Higuchi and Korsmeier-Peppas kinetic model (the value of n obtained in the Korsmeier-Peppas kinetic model for both pH is approximately equal to 0.5), the rate constant values in both models are approximately equal. As a result, Fickian diffusion is effective in drug release. According to Kopcha's kinetic model and the values of A and B obtained by fitting the release data, the A/B ratio for pH 5 and 7.4 was 9.77 and 6.73, respectively, since this ratio is greater than 1, it indicates that is that the release mechanism for both pHs is more influenced by diffusion.

**Table 1:** Kinetic parameters obtained from fitting experimental data with different kinetic models.

Release model	Parameter	pH = 5	pH = 7.4
Zero-order ($\div{C = k_{ \circ } t + C_{0}}$)	k˳ (h^−1^) R^2^ RMS	17.771 0.842 4.219	13.562 0.793 3.434
First order ($C = C_{ \circ } \left( {1 - Exp\left( { - kt} \right)} \right)$)	k (h^−1^) R^2^ RMS	1.518 0.972 1.764	1.490 0.930 2.116
Higuchi (M_t_/M͚ = k·t^1/2^)	k (h^−1/2^) R^2^ RMS	23.207 0.923 2.786	16.533 0.867 2.788
Korsmeier-Peppas (M_t_/M͚ = k·t^n^)	k (h^−n^) R^2^ RMS n	23.231 0.929 2.835 0.571	16.553 0.876 2.815 0.592
Kopcha (M = A·t^1/2^ + B·t)	A B R^2^ RMS	21.023 2.150 0.924 2.923	14.367 2.132 0.870 2.889

In the zero-order kinetic model, C is the drug concentration at time t, C_0_ is the initial concentration of the drug, t is the time, and K_0_ is the zero-order rate constant. In the first-order kinetic model, C_°_ is the initial concentration of the drug and K is the first-order rate constant. In the Higuchi kinetic model, M_t_ is the amount of drug released at time t, M_∞_ is the amount released at the infinite time, and k is Higuchi's speed constant. In the Korsmeier-Peppas kinetic model, t is the time, k is the Korsmeier-Peppas release rate constant, and n is the release exponent. In the Kopcha kinetic model, A and B correspond to the constants of diffusion rate and erosion rate, respectively.

### In-vitro cytotoxicity assay

**Figure 6:**
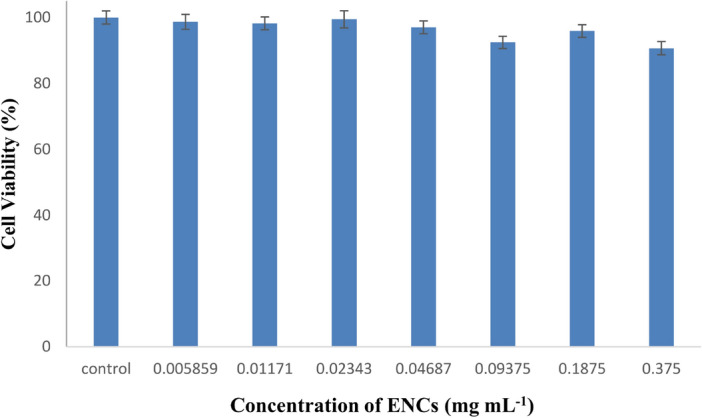
Cell viability of MCF7 after 24-h incubation with different concentrations of ENCs.

Figure 6 shows the cell survival percentage after exposing cells to the synthesized chitosan/alginate nanocarrier without any drug for 24 h. The control group with no nanocarrier exposure had 100% cell survival, indicating maximum cell viability. The groups treated with only the nanocarrier without any drug had almost constant cell survival rates close to 100%. This shows that the synthesized nanocarrier alone does not cause significant cell death and does not have significant toxicity effects on the studied cell line. The nanocarrier alone is considered biocompatible as it does not negatively impact cell viability. Any cell death observed when the nanocarrier contains a drug is likely due to the effect of the drug, not the nanocarrier itself.

**Figure 7:**
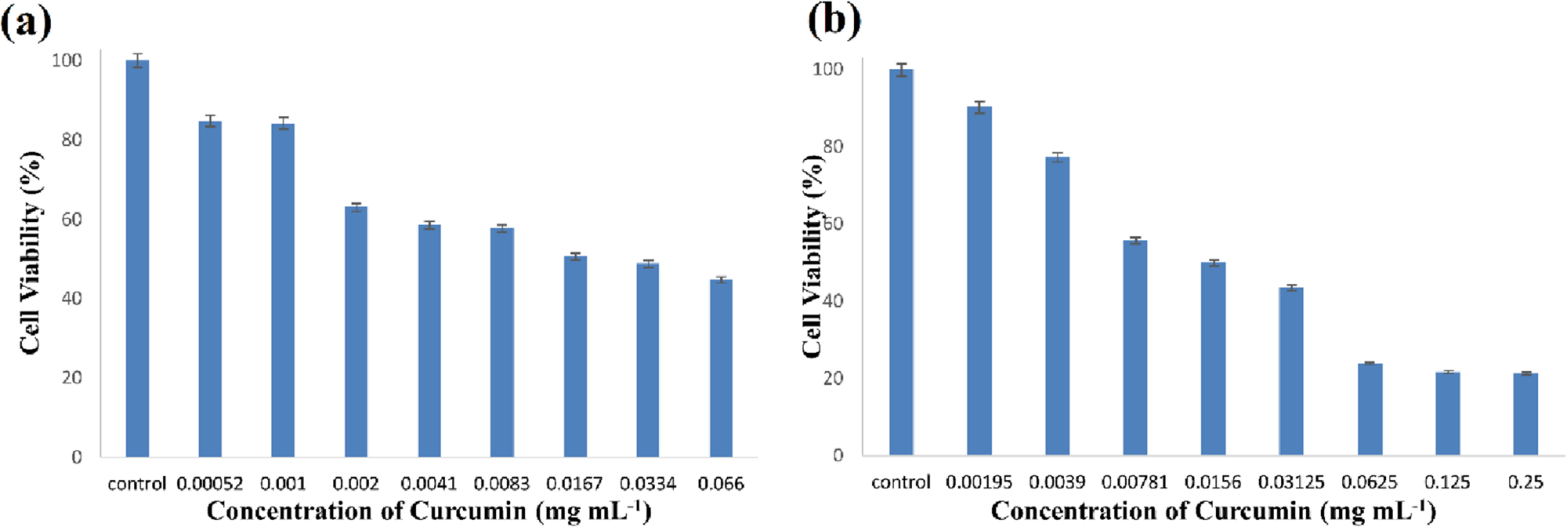
Cell viability of MCF7 after 4-h incubation with different concentrations of CurNCs (**a**) and curcumin (**b**).

Figure 7 shows the results of the MTT assay for nanocarrier containing the curcumin and pure curcumin in a period of 4 h. As it is clear from Fig. 6, as the concentration of nanocarrier and pure curcumin increases, the trend of the graphs decreases and the percentage of cell viability decreases, which indicates cell death. It means that the drug is released into the environment of the cells and causes cell death, and in fact, the percentage of cell survival decreases with the increase in the concentration of the drug. As Fig. 6 shows, cell death increases as the concentrations of CurNCs or curcumin increases. The value of IC_50_ (half maximal inhibitory concentration) for the nanocarrier containing curcumin and curcumin was calculated using GraphPad prism software and the values of 14.86 and 16.45 mg mL^−1^ were obtained, respectively. As a result, the required dose of the synthesized nanocarrier is comparable to curcumin for inducing cell death. Comparatively, CurNCs showed higher cell growth inhibition. Moreover, loading curcumin into the pH-sensitive nanocarrier allows it to be released more in the acidic environments of cancerous cells, potentially reducing side effects to other cells.

## Conclusions

In summary, a custom-fabricated setup was developed for the synthesis of drug nanocarriers. The setup utilizes individual pneumatic nebulizers for the production of precursor solution aerosols. Thus, the reaction between precursors occurs in the aerosol phase. This approach leads to decreasing the synthesis time and improving the feasibility of synthesizing sophisticated nanoparticles. The heater tunnel can act both as the reaction and desolvation zone. The temperature of the tunnel can be adjusted to increase the reaction rates. The number of nebulizers can be increased based on the flow design of the nanoparticles needed. Using the fabricated setup, CurNCs were successfully synthesized. The synthesized nanocarrier showed prolonged pH-dependent release behavior as the drug release was increased at acidic pH. Considering that the environment around cancerous cells is more acidic than normal cells, the synthesized nanocarrier could be promising for targeted delivery of its cargo to cancer cells. This leads to lower side effects of the cargo drug to the normal healthy cells.

## Data Availability

Experimental data will be available on request, please contact the corresponding author at madrakian@basu.ac.ir.
